# Old but gold – Antia and Buch chondrocutaneous advancement flap for helical reconstruction: a series of cases^☆^

**DOI:** 10.1016/j.abd.2021.01.010

**Published:** 2022-09-06

**Authors:** Paula Hitomi Sakiyama, Thiago Augusto Ferrari, Raíssa Rigo Garbin, Roberto Gomes Tarlé

**Affiliations:** a Department of Dermatology, Hospital Santa Casa de Curitiba, Curitiba, PR, Brazil; b Discipline of Dermatology, Pontifícia Universidade Católica do Paraná, Curitiba, PR, Brazil

Dear Editor,

The auricle or pinna is a frequent target of malignant neoplasms. Half of the tumors are located in the helix, and many defects involve both skin and cartilage, making surgical reconstruction a challenge.^1^ In this article, the authors describe three cases of skin neoplasms in the helix repaired with Antia and Buch chondrocutaneous advancement flap.^2^ Table 1 describes step-by-step the technique.

**Table 1:** Steps for performing Antia and Buch flap.

1) Antisepsis
2) Demarcation of the surgical margin
3) Local anesthesia and lesion excision
4) Drawing of the flap, showing a line at the limit anterior to the helix, inferior to the defect, extending to the lobe, and the retroauricular compensation triangle
5) Linear incision on the anterior surface of the ear, along the helix curvature up to the lobe, including the cartilage, without transfixing the retroauricular skin. A compensation triangle can be drawn on the lobe
6) Retroauricular detachment over the cartilage with blunt scissors (flap pedicle)
7) Hemostasis
8) Chondrocutaneous flap advancement through the cartilage suture
9) Resection of the retroauricular compensation triangle
10) Suture of the skin
11) Compressive dressing

The first patient presented an *in situ* squamous cell carcinoma (SCC), measuring 13 mm in its largest diameter, submitted to Mohs micrographic surgery (MMS) and removed in the first stage. The second patient had an infiltrative basal cell carcinoma (BCC), measuring 8 mm in its largest diameter, removed in 2 stages, using MMC. Finally, the third case was a well-differentiated SCC, measuring 10 mm in its largest diameter, resected using the conventional technique (Fig. 1).

**Figure 1:**
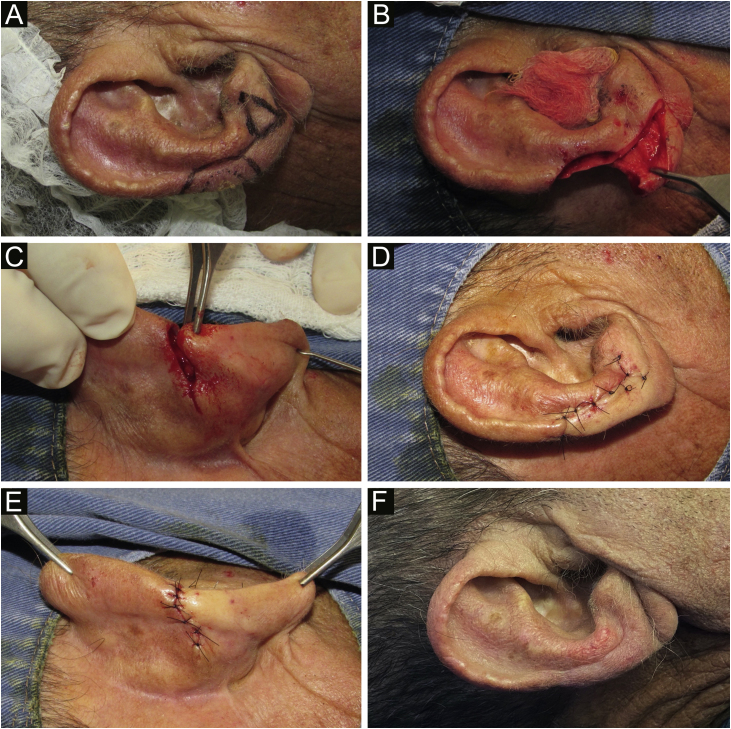
(A) Clinical lesion and margin delimitation. Design of the flap, with a compensation triangle at the level of the auricular lobe. (B) Composite surgical defect (skin and cartilage) and linear incision on the anterior surface of the ear. (C) Posterior view of the advancement movement, after resection of the retroauricular compensation triangle. (D‒E) Suture by planes and immediate postoperative period. (F) Late postoperative period.

Surgical planning is essential to attain good results in the management of tumors in the auricular region. Among its peculiarities, a significant amount of cartilage is present which may not resist distortions secondary to scar contracture. Additionally, there is an increased risk of necrosis due to the thinness of the auricular skin. In the case of the helix, the choice of repair is based on the location, size of the defect, and the amount of cartilage loss. Small defects (<1‒1.5 cm) are easily reconstructed with wedge excision and primary closure, unlike larger defects, which require the use of grafts or flaps.

Grafts require a preserved cartilaginous structure, with viable perichondrium or cartilage perforations to allow irrigation through the contralateral bed. In composite defects, the structural loss of the helix margin requires cartilage and skin for its repair, which can be performed using the following techniques: composite graft from the contralateral auricle; interpolated flaps using cartilage; chondrocutaneous flap.^1^, ^3^, ^4^, ^5^ The latter is frequently used and can be performed through a skin incision on only one surface of the ear and cartilage, as described by Antia and Buch, or through a full-thickness incision including the skin of the two surfaces and cartilage (Fig. 2).

**Figure 2:**
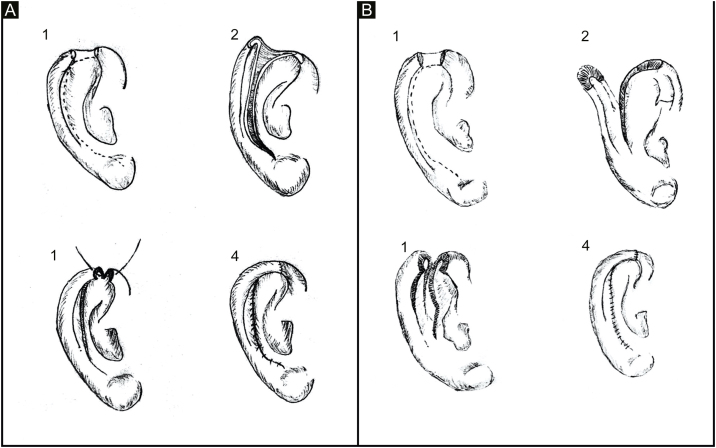
(A) Classic chondrocutaneous flap (A.1) Helical defect, dashed lines indicate skin and cartilage incisions. (A.2) Incision on the anterior surface of the ear, including the cartilage, without transfixing the retroauricular skin. (A.3) Advancement movement and cartilage suturing. (A.4) Skin suturing. (B) Full-thickness chondrocutaneous flap. (B.1) Helical defect. (B.2) Incision on the anterior and posterior surface of the ear, including cartilage, transfixing the retroauricular skin. (B.3) Advancement movement. (B.4) Suture by planes.

The chondrocutaneous flap was first described by Antia and Buch, in 1967. The principle of the procedure is to advance the skin and cartilage of the intact portion of the helix adjacent to the defect, based on a wide and secure retroauricular pedicle, with greater preservation of the vascularization of the posterior auricular artery.^2^ However, the classic technique has lost prominence since the initial report and several modifications have been made ‒ including the incision of the transition line from the helix to the dorsum of the ear, limiting irrigation by restricting the pedicle to the lower portion of the flap only, which carries the risk of local vascularization impairment.

Therefore, Antia and Buch flap, despite having been described more than 50 years ago, maintains its role among the techniques of helix reconstruction, and its knowledge is important for dermatological surgeons. It is an effective and safe method, performed in a single step with the additional advantage of preserving local vascularization, reducing the risk of necrosis.

## Financial support

None declared.

## Authors' contributions

Paula Hitomi Sakiyama: Approval of the final version of the manuscript; design and planning of the study; drafting and editing of the manuscript; collection, analysis, and interpretation of data; critical review of the literature; critical review of the manuscript.

Thiago Augusto Ferrari: Approval of the final version of the manuscript; design and planning of the study; drafting and editing of the manuscript; collection, analysis, and interpretation of data; critical review of the literature; critical review of the manuscript.

Raíssa Rigo Garbin: Approval of the final version of the manuscript; design and planning of the study; collection, analysis, and interpretation of data; effective participation in research orientation; intellectual participation in the propaedeutic and/or therapeutic conduct of studied cases; critical review of the literature; critical review of the manuscript.

Roberto Gomes Tarlé: Approval of the final version of the manuscript; design and planning of the study; effective participation in research orientation; intellectual participation in the propaedeutic and/or therapeutic conduct of the studied cases; critical review of the literature; critical review of the manuscript.

## Conflicts of interest

None declared.
